# Correction: Understanding mono- and bi-metallic Au and Ni nanoparticle responses to fast heating

**DOI:** 10.1039/d4na90099e

**Published:** 2024-09-23

**Authors:** Tatiana E. Itina

**Affiliations:** a Université Jean Monnet Saint-Etienne, CNRS, Institut d Optique Graduate School, Laboratoire Hubert Curien UMR 5516 F-42023 Saint-Etienne France tatiana.itina@univ-sjlvt-etienne.fr

## Abstract

Correction for ‘Understanding mono- and bi-metallic Au and Ni nanoparticle responses to fast heating’ by Tatiana E. Itina *et al.*, *Nanoscale Adv.*, 2024, https://doi.org/10.1039/d4na00634h.

The author regrets that eqn (4) contains errors in its notation and so requires correction.

The corrected equation is as follows.4
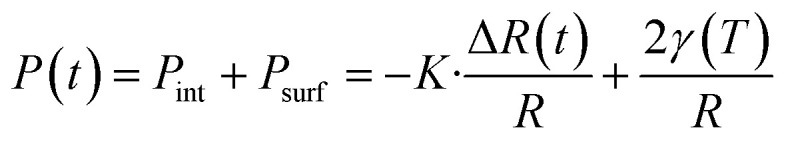


As a result of this error, a correction should also be made to the reference to eqn (4) in the following sentence:

«The basic equation for the oscillations of the radius *r*(*t*) is:»

Should be:

«The basic equation for the oscillations of the radius *R*(*t*) is:»

The Royal Society of Chemistry apologises for these errors and any consequent inconvenience to authors and readers.

